# Comparison of the Abbott Alinity m and Qiagen Artus assays for the quantification of BK virus in clinical samples

**DOI:** 10.1016/j.ijregi.2024.100504

**Published:** 2024-12-16

**Authors:** Honorine Fenaux, Eric Marchadier, Alexandra Champagne, Corinne Prégermain, Lina Mouna

**Affiliations:** Virology Department, Hôpital Paul Brousse, INSERM U1193, AP-HP, Université Paris Saclay, Paris, France

**Keywords:** BK virus, Kidney transplant recipients, Viral load, Alinity m, Interstitial nephropathy

## Abstract

**Objectives:**

BK virus (BKV) is highly seroprevalent in humans. After primary infection, it remains latent in the urinary tract and can reactivate in immunocompromised individuals, leading to interstitial nephropathy or hemorrhagic cystitis. The BKV viral load (VL) in plasma correlates with the occurrence of nephropathy and can be monitored in kidney graft recipients; the early detection of BKV viremia can enable an early reduction of immunosuppressant drug doses and the prevention of BKV-associated nephropathy. BKV VL is measured using real-time reverse transcription-polymerase chain reaction. Our aim was to evaluate the Abbott Alinity m BKV AMPL kit versus the Artus BKV quantification kit (Qiagen, Hilden, Germany).

**Methods:**

We compared the methods on 69 plasma samples, 54 urine samples, and 12 external quality control samples. To assess analytical sensitivity, serial dilutions of internal control were also tested.

**Results:**

Specificity reached 100%. Fifty-seven of the 59 plasma samples were found to be positive (sensitivity: 96.6%), as were 46 of the 48 urine samples (sensitivity: 95.8%). In quantifiable samples, the VL was correlated with the findings on plasma (r = 0.965) and urine samples (r = 0.971). The mean differences were 0.78 log_10_ and 0.28 log_10_ in plasma and urine samples, respectively. A comparison of the BKV VL in external quality control samples produced a correlation coefficient of 0.930 and a mean difference of 0.07 log_10_ IU/ml. The calculated analytical sensitivity was 15 IU/ml with 95% probability.

**Conclusions:**

The Alinity m BKV AMPL kit is compatible with diagnostic use and can be used in a “random-access” manner, thus reducing the time between sampling and the results being available to clinicians.

## Highlights

• Monitoring of BK virus (BKV) viral load helps prevent BKV-associated nephropathy
• The Abbott Alinity m BKV AMPL kit is suitable for use in a diagnostic context
• The Alinity m platform allows testing in a “random-access” manner

## Background

BK virus (BKV) belongs to the *Polyomaviridae* family and has a high rate of worldwide adult seroprevalence at approximately 80%. After primary infection, which happens most frequently during childhood, the virus remains latent in the urinary tract [1]. In immunocompromised individuals, it can reactivate and lead to interstitial nephropathy (kidney graft recipients) [2] or hemorrhagic cystitis (hematopoietic stem cell recipients) [3]. BKV-associated nephropathy can ultimately lead to graft loss.

The BKV plasmatic viral load (VL) correlates with the occurrence of nephropathy, especially with values >10,000 copies/ml for 28 days [2]. BKV can be excreted in the urine, even in asymptomatic immunocompetent individuals. BKV viruria is not correlated with nephropathy [4] or hemorrhagic cystitis, but the presence of BKV in the urine of an immunocompromised patient, particularly with a high VL, can suggest that the virus might be reactivated and that monitoring of the blood is warranted. There is no specific antiviral treatment for BKV reactivation, but it is possible to lower the doses of immunosuppressant drugs, which may help to control BKV reactivation and prevent BKV-associated nephropathy [4]. The sooner the immunosuppressant drug doses are lowered, the better the control of BKV reactivation: the monitoring of BKV VL is important to detect any reactivation before the occurrence of nephropathy and graft malfunction.

BKV VL is measured using real-time reverse transcription-polymerase chain reaction (RT-PCR).

A BKV quantification kit has recently been developed on the Alinity m platform (Abbott Molecular, Des Plaines, IL, USA); this would enable “random-access” processing and the timely delivery of biologic results compared with the many platforms that operate on a “batch” basis.

## Objectives

Our aim was to compare the Abbott Alinity m BKV quantification kit versus the Artus BKV quantification kit (Qiagen, Hilden, Germany).

## Study design

### Reference method

BKV quantitative RT-PCR assays were performed on plasma and urine samples for diagnostic purposes using the QIAsymphony/RotorGeneQ (Qiagen); this was considered as the reference method. Nucleic acid was extracted using the QIAsymphony DSP Virus/Pathogen Midi Kit and BKV quantitative RT-PCR with the Artus® BKV-QS-RGQ kit (Qiagen). The results were expressed in copies/ml (and log_10_ copies/ml) according to the manufacturer's instructions. The limits of detection and quantification were 200 copies/ml – 2.30 log_10_ copies/ml. The samples were then stored at –80°C.

### Comparison of methods

We tested 69 plasma samples and 54 urine samples on the Alinity m platform using the Alinity m BKV AMPL kit. Urine samples were diluted with the Alinity m Urine Transport kit according to the manufacturer's instructions. The results were expressed in IU/ml (and log_10_ IU/ml). The limit of detection was 50 IU/ml – 1.7 log_10_ IU/ml, and the limits of quantification were 50 IU/ml – 1.7 log_10_ IU/ml and 1,000,000,000 IU/ml – 9 log_10_ IU/ml, according to the manufacturer.

In the event of any discrepancies between the two methods, the procedure was repeated using both of them.

We tested 15 external quality control (EQC) samples (Quality Controls for Molecular Diagnostics, QCMD, Glasgow, United Kingdom), for which “reference” values expressed in IU/ml were available, which thus enabled us to compare them using the same units as Alinity m.

### Sensitivity

To assess analytical sensitivity, an Alinity m BKV CTRL Kit (09N85-080) was used. Serial dilutions of low positive control (2.32-3.82 log_10_ IU/ml) and high positive control (5.40-6.90 log_10_ IU/ml) were tested. According to Abbott kit instructions, concentrations were standardized against the 1st World Health Organization International Standard for BK Virus DNA (NIBSC code: 14/212). The low positive internal control was diluted in a negative urine sample (in triplicate 1:10, 1:100, 1:1000, and 1:10,000). The high positive internal control was also diluted in a negative urine sample (in duplicate 1:10, 1:100, 1:1000, 1:10,000; in quadruplicate 1:30,000; in triplicate 1:50,000).

### Repeatability and reproducibility

Repeatability and reproducibility were assessed as a first step using positive plasma and urine samples tested three times on the same day and twice more on two different days. As a second step, a diluted plasma sample and a diluted urine sample (dilution in negative plasma and urine samples, respectively) were tested 10 times on the same day (repeatability) and five times on day 1, five times on the morning of day 2, five times in the afternoon of day 2, and five times on day 3 (reproducibility).

### Statistics

The VLs obtained with the two kits were compared using Analyse-it v5.4 software.

## Results

### Comparison of methods

The results obtained for all samples can be found in Supplementary data (Tables S1 and S2).

All 10 plasma samples that were negative with the reference method also tested negative with the Alinity m kit. Of the 59 positive plasma samples, 57 were found to be positive (sensitivity: 96.6%), whereas the other two samples were low positive (<3 log_10_ copies/ml) (Table 1).

**Table 1:** Results of plasma samples when comparing the methods.

		Alinity m (log_10_ IU/ml)			
	*Not detected*	Not detected	Low positive	Positive	Total
**Artus (log_10_ copies/ml)**		10	0	0	10
	Low positive	2^a^	15	0	17
	Positive	0	14	28	42
	Total	12	29	28	69

a Low positive (<3 log_10_ copies/ml).

The six negative urine samples also tested negative with the Alinity m kit. Of the 48 positive urine samples, 46 were positive (sensitivity: 95.8%), whereas the other two samples were low positive (<3 log_10_ copies/ml) (Table 2).

**Table 2:** Results of urine samples when comparing the methods.

		Alinity m (log_10_ IU/ml)			
	*Not detected*	*Not detected*	Low positive	Positive	Total
**Artus (log_10_ copies/ml)**		6	0	0	6
	Low positive	2^a^	12	0	14
	Positive	0	2	32	35
	Total	8	14	32	54

a Low positive (<3 log_10_ copies/ml).

**Figure 1:**
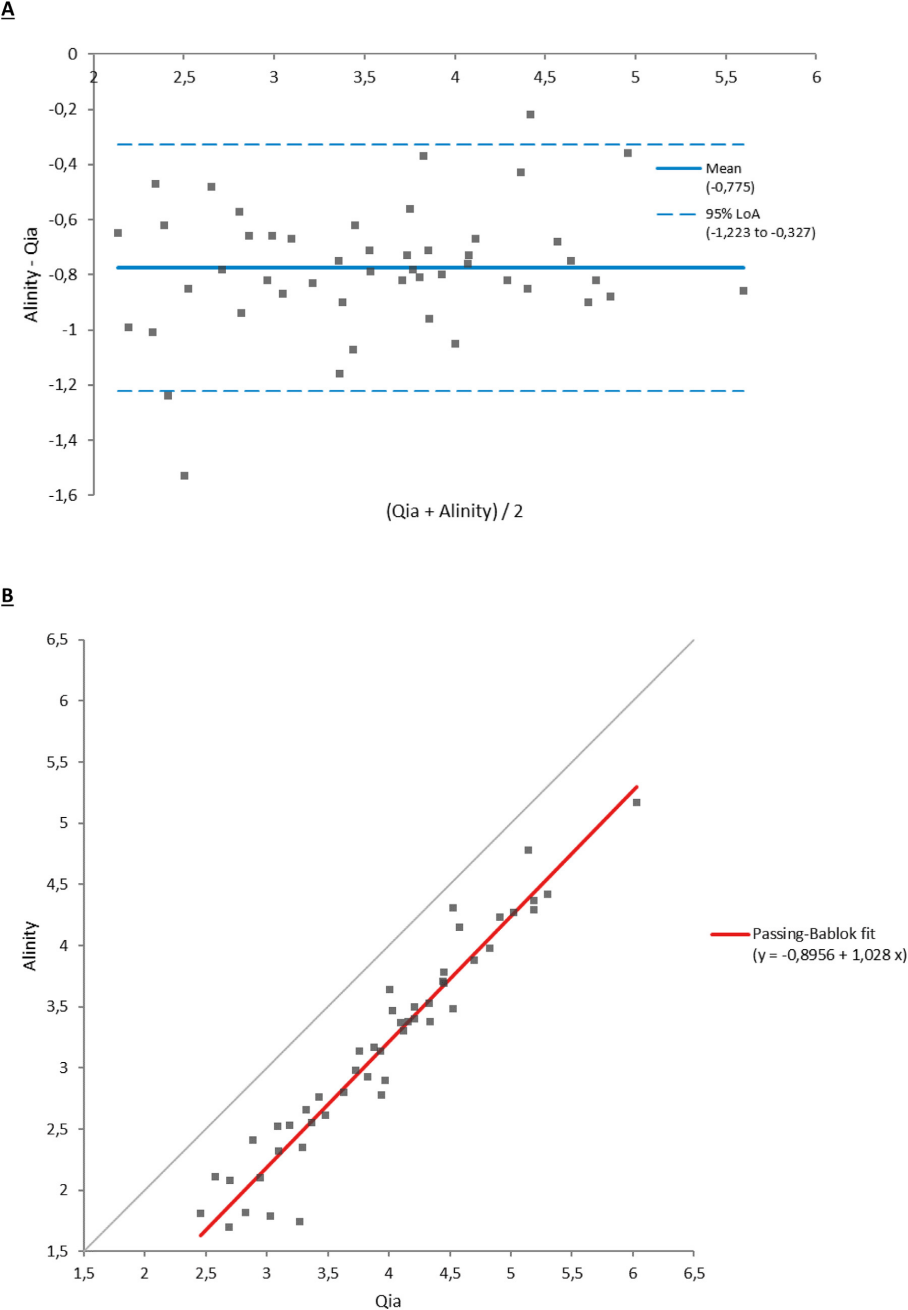
Comparison of viral loads in plasma samples between the reference method (Qia) and the tested method (Alinity). (a) Bland-Altman test. (b) Passing-Bablok test.

**Figure 2:**
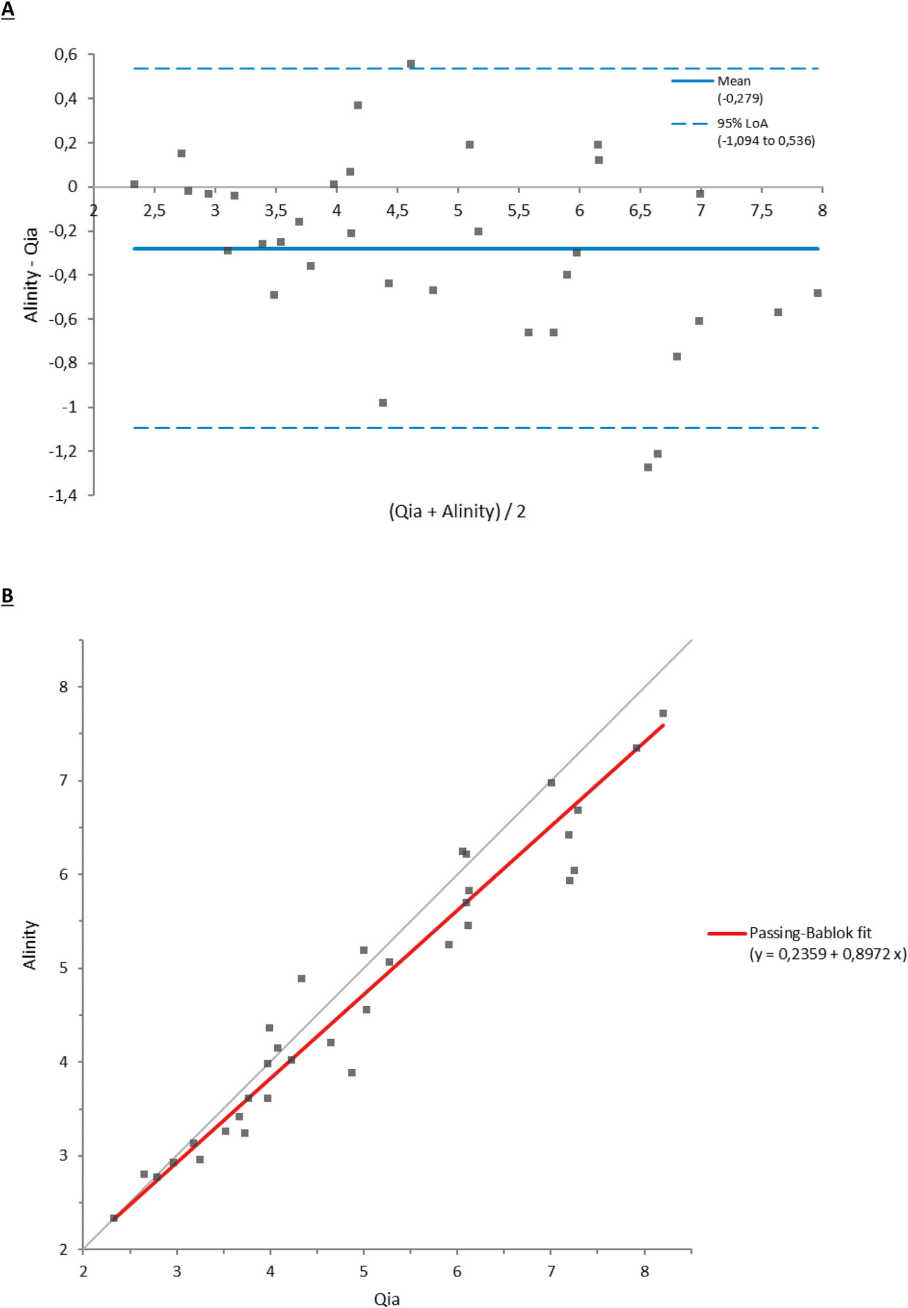
Comparison of viral loads in urine samples between the reference method (Qia) and the tested method (Alinity). (a) Bland-Altman test. (b) Passing-Bablok test.

In quantifiable samples, VLs were correlated in both plasma (r = 0.965) and urine samples (r = 0.971) (Figure 1, Figure 2). The mean differences were 0.78 log_10_ and 0.28 log_10_ in plasma and urine samples, respectively.

**Figure 3:**
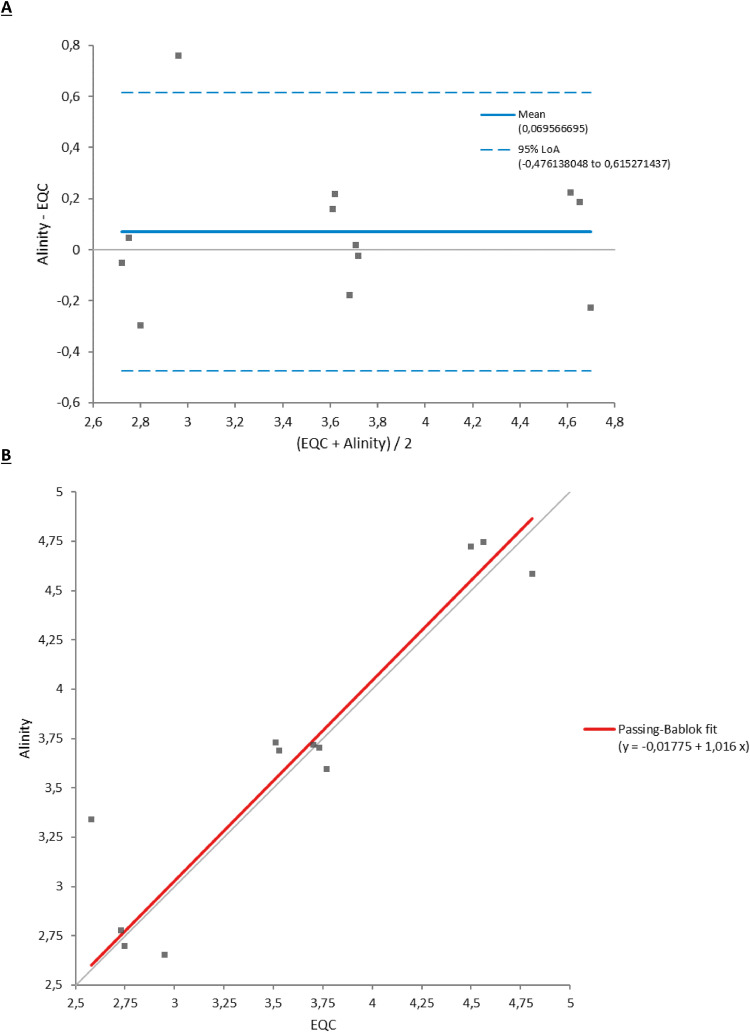
Comparison of viral loads in EQC samples between their consensus values (supplied by the QCMD organization) and those obtained with the Alinity test. (a) Bland-Altman test. (b) Passing-Bablok test. EQC, external quality control.

The comparison of BKV VL in EQC samples is shown in Table 3 and Figure 3; r = 0.930, the mean difference being 0.07 log_10_ IU/ml.

**Table 3:** Results of external quality control samples.

		Alinity m (log_10_ IU/ml)			
		*Not detected*	Low positive	Positive	Total
Quality Controls for Molecular Diagnostics, **QCMD**, Glasgow, United Kingdom (log_10_ IU/ml)	*Not detected*	3	0	0	3
	Low positive	0	3	1	4
	Positive	0	0	8	8
	Total	3	3	9	15

### Sensitivity

The probit analysis showed that a VL of 15 IU/ml could be detected with a 95% probability (probit analysis, *P* < 0.0001) (Supplementary data S3).

### Repeatability and reproducibility

Inter-assay and intra-assay coefficients of variation were assessed in two steps, as explained in the study design.

Inter-assay coefficients of variation:

In plasma, the coefficient of variation varies between 0.40% (highest tittered sample) and 1.70% (lowest tittered samples).
In urine samples, the coefficient of variation varies between 0.38% (highest tittered sample) and 3.94%.

Intra-assay coefficients of variation:

In plasma, the coefficient of variation varies between 0.80% and 1.19% (lowest tittered samples).
In urine samples, the coefficient of variation varies between 0.43% (highest tittered sample) and 2.20% (Table 4).

**Table 4:** Results of repeatability and reproducibility assays.

VL = viral load.

## Discussion

We tested a method for the quantification of BKV VL using an Abbott Alinity m platform. The results showed a high correlation, especially with those obtained on EQC, for which a “reference” value in IU/ml was available; a variation of less than 0.5 log_10_ was not considered significant.

The analytical sensitivity declared by the manufacturer is 50 IU/ml, which is higher than what we found (15 IU/ml). However, these very low VL values are not correlated with BKV-associated nephropathy; thus this finding would make no difference in terms of follow-up.

Repeatability and reproducibility vary among samples, particularly urine samples, but are still within the permitted and recognized limits. Of note, urine samples need to be diluted to obtain the required VL, which might have had consequences in terms of repeatability and reproducibility and could partly explain the results. In the context of molecular biology methods, a difference of less than 0.5 log_10_ is not considered to be significant; the probability of such an event occurring is 5% when the coefficient of variation is 40% [5].

The present study had several limitations, such as a modest number of samples. Further studies are needed to complete our results. In addition, developing novel biomarkers for the diagnosis of BKV-associated nephropathy, such as the measurement of virus-specific T cell level and the development of cellular therapy, is ongoing and will be complementary to BKV VL tests. The Alinity m platform is practical, and assays are already available on that platform for viruses other than BKV: hepatitis B and C viruses, human immunodeficiency virus, and human papillomaviruses. It can, therefore, be applied to test a range of viruses, which could serve as a criterion for choosing between different methods. The Alinity m platform enables “random-access,” unlike our reference method: Qiagen platform operates on a “batch” mode with little flexibility, which may cause delayed availability of results while pending a sufficient number of samples to complete a batch to avoid increased costs. The Alinity m platform is a fully automated “random-access” system with a time-to-first result of less than 115 minutes and a throughput of up to 300 samples in 8 hours. That allows for rapid time-to-first results for the benefit of patients. The potential of this platform is to significantly improve workflow efficiency in diagnostic laboratories.

To conclude, the Alinity m BKV AMPL kit is compatible with diagnostic use and can be used in a “random-access” manner, thus reducing the time between sampling and results being available to clinicians.

## Declarations of competing interest

The authors have no competing interest to declare.

## Appendix Supplementary materials

Image, application 1

Image, application 2

## Footnotes

Supplementary material associated with this article can be found, in the online version, at doi:10.1016/j.ijregi.2024.100504.
