# Supplementary material for: Comparison of the Abbott Alinity m and Qiagen Artus assays for the quantification of BK virus in clinical samples
Source: IJID Reg. 2024 Dec 16;14:100504. doi: 10.1016/j.ijregi.2024.100504 (PMC11745809; doi:10.1016/j.ijregi.2024.100504)
Supplement: Supplementary file 2 [file mmc2.docx]

Table S1 : Plasma

| Sample | Artus (log_10_ copies/ml) | Alinity m (log_10_ IU/ml) |
| --- | --- | --- |
| 1 | 3.10 | 2.32 |
| 2 | 3.73 | 2.98 |
| 3 | 6.03 | 5.17 |
| 4 | 5.14 | 4.78 |
| 5 | 3.19 | 2.53 |
| 6 | 4.83 | 3.98 |
| 7 | 4.58 | 4.15 |
| 8 | 4.10 | 3.37 |
| 9 | 4.12 | 3.3 |
| 10 | 3.09 | 2.52 |
| 11 | 3.43 | 2.76 |
| 12 | 4.01 | 3.64 |
| 13 | 3.32 | 2.66 |
| 14 | 3.63 | 2.8 |
| 15 | 2.69 | 1.7 |
| 16 | 3.03 | 1.79 |
| 17 | 3.27 | 1.74 |
| 18 | 4.70 | 3.88 |
| 19 | 3.83 | 2.93 |
| 20 | 3.97 | 2.9 |
| 21 | 4.21 | 3.4 |
| 22 | 3.48 | 2.61 |
| 23 | 4.45 | 3.78 |
| 24 | 3.88 | 3.17 |
| 25 | <2.30 | <1.7 |
| 26 | 4.33 | 3.53 |
| 27 | 4.91 | 4.23 |
| 28 | 2.95 | 2.1 |
| 29 | 2.70 | 2.08 |
| 30 | 2.62 | <1.7 |
| 31 | <2.30 | <1.7 |
| 32 | 3.94 | 2.78 |
| 33 | 4.53 | 4.31 |
| 34 | 2.74 | <1.7 |
| 35 | 4.44 | 3.71 |
| 36 | 3.93 | 3.14 |
| 37 | 2.89 | 2.41 |
| 38 | 3.76 | 3.14 |
| 39 | 4.03 | 3.47 |
| 40 | 2.83 | 1.82 |
| 41 | 4.21 | 3.5 |
| 42 | 4.16 | 3.38 |
| 43 | 4.34 | 3.38 |
| 44 | 5.19 | 4.37 |
| 45 | 2.46 | 1.81 |
| 46 | 4.45 | 3.69 |
| 47 | 2.38 | <1.7 |
| 48 | 5.19 | 4.29 |
| 49 | 5.02 | 4.27 |
| 50 | 4.53 | 3.48 |
| 51 | 3.29 | 2.35 |
| 52 | <2.30 | <1.7 |
| 53 | 3.37 | 2.55 |
| 54 | 2.48 | 1.72 |
| 55 | <2.30 | Undetectable |
| 56 | <2.30 | <1.7 |
| 57 | <2.30 | Undetectable |
| 58 | 5.30 | 4.42 |
| 59 | 2.58 | 2.11 |

Table S2 : Urine

| Sample | Artus (log_10_ copies/ml) | Alinity m (log_10_ IU/ml) | 2nd time on Artus | 2nd time on Alinity m |
| --- | --- | --- | --- | --- |
| 1 | 7.19 | 6.42 |  |  |
| 2 | <2.30 | 2.05 |  |  |
| 3 | 6.40 | <1.7 | Undetectable | Undetectable |
| 4 | 6.06 | 6.25 |  |  |
| 5 | 7.92 | 7.35 |  |  |
| 6 | 7.01 | 6.98 |  |  |
| 7 | 9.26 | >9 |  |  |
| 8 | 4.87 | 3.89 |  |  |
| 9 | 3.97 | 3.61 |  |  |
| 10 | 8.20 | 7.72 |  |  |
| 11 | 2.33 | 2.34 |  |  |
| 12 | 2.79 | 2.77 |  |  |
| 13 | 9.96 | >9 |  |  |
| 14 | 3.77 | 3.61 |  |  |
| 15 | 4.23 | 4.02 |  |  |
| 16 | 2.46 | <1.7 |  |  |
| 17 | <2.30 | 1.89 |  |  |
| 18 | 3.97 | 3.98 |  |  |
| 19 | 7.25 | 6.04 |  |  |
| 20 | 3.18 | 3.14 |  |  |
| 21 | <2.30 | 2.56 |  |  |
| 22 | <2.30 | Undetectable |  |  |
| 23 | 2.96 | 2.93 |  |  |
| 24 | 9.15 | >9 |  |  |
| 25 | 5.00 | 5.19 |  |  |
| 26 | 5.27 | 5.07 |  |  |
| 27 | 3.25 | 2.96 |  |  |
| 28 | 6.12 | 5.46 |  |  |
| 29 | 2.65 | 2.8 |  |  |
| 30 | 7.29 | 6.68 |  |  |
| 31 | <2.30 | <1.7 |  |  |
| 32 | 4.65 | 4.21 |  |  |
| 33 | 5.91 | 5.25 |  |  |
| 34 | 3.99 | 4.36 |  |  |
| 35 | <2.30 | <1.7 |  |  |
| 36 | 3.94 | 5.7 | 6.1 | 5.42 |
| 37 | 9.15 | >9 |  |  |
| 38 | 3.52 | 3.26 |  |  |
| 39 | 6.10 | 6.22 |  |  |
| 40 | <2.30 | 2.12 |  |  |
| 41 | 4.33 | 4.89 |  |  |
| 42 | 5.03 | 4.56 |  |  |
| 43 | <2.30 | <1.7 |  |  |
| 44 | 4.08 | 4.15 |  |  |
| 45 | 3.67 | 3.42 |  |  |
| 46 | 6.13 | 5.83 |  |  |
| 47 | 3.73 | 3.24 |  |  |
| 48 | <2.30 | Undetectable |  |  |
